# Cattle select African savanna termite mound patches less when sharing habitat with wild herbivores

**DOI:** 10.1002/ece3.4452

**Published:** 2018-08-19

**Authors:** Wilfred O. Odadi, Grace K. Charles, Truman P. Young

**Affiliations:** ^1^ Department of Natural Resources Egerton University Egerton Kenya; ^2^ Mpala Research Centre Nanyuki Kenya; ^3^ Department of Plant Sciences University of California, Davis Davis California

**Keywords:** competition, grazing facilitation, livestock–wildlife interactions, nutrient‐rich foraging hotspots, *Odontotermes*, resource selection

## Abstract

African savanna termite mounds function as nutrient‐rich foraging hotspots for different herbivore species, but little is known about their effects on the interaction between domestic and wild herbivores. Understanding such effects is important for better management of these herbivore guilds in landscapes where they share habitats. Working in a central Kenyan savanna ecosystem, we compared selection of termite mound patches by cattle between areas cattle accessed exclusively and areas they shared with wild herbivores. Termite mound selection index was significantly lower in the shared areas than in areas cattle accessed exclusively. Furthermore, cattle used termite mounds in proportion to their availability when they were the only herbivores present, but used them less than their availability when they shared foraging areas with wild herbivores. These patterns were associated with reduced herbage cover on termite mounds in the shared foraging areas, partly indicating that cattle and wild herbivores compete for termite mound forage. However, reduced selection of termite mound patches was also reinforced by higher leafiness of *Brachiaria lachnantha* (the principal cattle diet forage species) off termite mounds in shared than in unshared areas. Taken together, these findings suggest that during wet periods, cattle can overcome competition for termite mounds by taking advantage of wildlife‐mediated increased forage leafiness in the matrix surrounding termite mounds. However, this advantage is likely to dissipate during dry periods when forage conditions deteriorate across the landscape and the importance of termite mounds as nutrient hotspots increases for both cattle and wild herbivores. Therefore, we suggest that those managing for both livestock production and wildlife conservation in such savanna landscapes should adopt grazing strategies that could lessen competition for forage on termite mounds, such as strategically decreasing stock numbers during dry periods.

## 1 INTRODUCTION

Tropical savannas cover approximately 20% of the earth's land surface area, and occur more extensively in Africa than in any other continent (Scholes & Archer, 1997). In Africa, these savannas occur exclusively in the sub‐Saharan region where they cover approximately 50% of the total land area (Du Toit & Cumming, 1999; Parr, Lehmann, Bond, Hoffmann, & Andersen, 2014). The African savanna biome supports the greatest diversity of native ungulates on earth, much of which is concentrated in East Africa (Du Toit & Cumming, 1999; Lorenzen, Heller, & Siegismund, 2012; Ripple et al., 2015; Turpie & Crowe, 1994). This high ungulate diversity is related to the high spatial vegetation heterogeneity (Davies, Baldeck, & Asner, 2016; Du Toit & Cumming, 1999; Lorenzen et al., 2012), which enhances resource partitioning and coexistence among sympatric ungulate species. In addition to serving as a critical biodiversity reservoir, African savannas also support extensive livestock production through pastoralism and commercial ranching. Wild and domestic herbivores share habitats in these savannas, especially on unfenced communal, private, and public lands.

Vegetation heterogeneity in African savannas is shaped by many abiotic and biotic factors operating at multiple spatial scales (Pickett, Cadenasso, & Benning, 2003; Scholes & Archer, 1997). At regional to landscape scales, precipitation, fire, and herbivory are the principal drivers of savanna vegetation structure and composition (Asner et al., 2009; Bond, Woodward, & Midgley, 2005; Sankaran et al., 2005). Within landscapes, variation in topography and soil characteristics becomes important factors contributing to vegetation heterogeneity (Baldeck et al., 2014; Venter, Scholes, & Eckhardt, 2003). At fine spatial scales, savanna heterogeneity is induced by the activities of earth‐burrowing invertebrates such as termites (Dangerfield, McCarthy, & Ellery, 1998; Gosling, Cromsigt, Mpanza, & Olff, 2012; Jouquet, Traoré, Choosai, Hartmann, & Bignell, 2011; Moe, Mobaek, & Narmo, 2009; Pringle, Doak, Brody, Jocque′, & Palmer, 2010; Sileshi, Arshad, Konaté, & Nkunika, 2010), and “glades” derived from abandoned livestock corrals (Augustine, 2003; Veblen, 2012; Young, Patridge, & Macrae, 1995).

Termites are regarded as major ecological engineers, and their mounds are common features in tropical savanna landscapes. Through their mound‐building and foraging activities, termites induce spatial heterogeneity by enhancing soil chemistry and texture, which in turn triggers cascading effects on the savanna biota and ecosystem processes. In particular, termites enhance litter decomposition, transport of soil material from deep horizons to the surface (thereby altering soil texture), redistribution and recycling of soil nutrients, and soil water infiltration (Brody, Palmer, Fox‐Dobbs, & Doak, 2010; Evans, Dawes, Ward, & Lo, 2011; Meyer, Braack, Biggs, & Ebersohn, 1999; Sileshi et al., 2010). These termite‐driven soil enhancements, coupled with other ecological disturbances such as herbivory and fire, create nutrient hotspots for both plants and other animal species (Buitenwerf, Stevens, Gosling, & Anderson, 2011; Dangerfield et al., 1998; Fox‐Dobbs, Doak, Brody, & Palmer, 2010; Sileshi et al., 2010; Stock, Bond, & van de Vijver, 2010). In African savannas, this ecosystem engineering is performed by diverse assemblages of fungus‐growing termites (subfamily Macrotermitinae, order Blattodea [Isoptera]). The most widespread of these termites belong to the genera *Odontotermes*,* Microtermes* and *Macrotermes* (Aanen & Eggleton, 2005; Bagine, 1984; Dangerfield et al., 1998; Gosling et al., 2012; Kooyman & Onck, 1987; Okwakol, 1992).

Because of termite‐driven soil enrichment, vegetation on termite mounds usually contains higher nutrient concentrations compared to vegetation in the surrounding matrix (Brody et al., 2010; Fox‐Dobbs et al., 2010; Muvengwi, Ndagurwa, Nyenda, & Mlambo, 2014). Mammalian herbivores generally forage selectively across the landscape in response to spatial and temporal heterogeneity, selecting nutrient‐rich patches that enable them to maximize nutrient intake while minimizing energetic costs of foraging and predation risk (Hopcraft, Olff, & Sinclair, 2010; Owen‐Smith, 2002). Termite mounds serve as such nutrient hotspots, typically preferred by mammalian herbivores (Brody et al., 2010; Cromsigt & te Beest, 2014; Davies, Levick, et al., 2016; Davies, van Rensburg, et al., 2016; Davies et al., 2014; Levick, Asner, Kennedy‐Bowdoin, & Knapp, 2010; Loveridge & Moe, 2004; Mobæk, Narmo, & Moe, 2005; Muvengwi, Witkowski, Daves, & Parrini, 2017). However, for ungulates, the degree of preference for termite mounds may vary among species depending on feeding habits, body size and associated dietary requirements, and digestive physiology (Anderson et al., 2016; Demment & Van Soest, 1985). In general, smaller, selective feeders should be more attracted to such nutritional hotspots than larger generalist feeders (Anderson et al., 2016). The general attractiveness of savanna termite mound vegetation patches to large herbivores can increase grazing pressure in these areas, which can potentially alter herbivore foraging patterns and influence interactions among different herbivore species or guilds.

Domestic and wild herbivores in African savannas do not share a long evolutionary history, but do share contemporary habitats, and as a result can exhibit high levels of niche overlap (Voeten & Prins, 1999). Niche overlap between domestic and wild herbivores would be expected to be greater on nutrient‐rich foraging patches such as termite mounds, which could potentially alter the direction and magnitude of their interaction. We have previously demonstrated in this system that livestock and wild herbivores largely compete with each other (Kimuyu et al., 2017; Odadi, Karachi, Abdulrazak, & Young, 2011; Odadi et al., 2017; Young, Palmer, & Gadd, 2005) but at times facilitate each other (Kimuyu et al., 2017; Odadi et al., 2011). These interactions are shaped by the effects of herbivory, fire, and rainfall on the vegetation. However, little is known about the effects of termite mounds as foraging hotspots on these interactions. Understanding such effects is pertinent to the management of both herbivore guilds and their shared habitats.

Here, we assessed the effects of shared foraging with native ungulates on cattle preference for termite mounds as foraging patches in a semiarid savanna rangeland in central Kenya. Our primary aim was to establish whether sharing habitat with wild mammalian herbivores alters the extent to which cattle select termite mounds as foraging patches. We compared selection of termite mound patches by cattle between areas from which wild herbivores had been experimentally excluded and areas cattle shared with these herbivores. We hypothesized that cattle would select termite mounds less when they share foraging areas with wild herbivores than when they access foraging areas exclusively.

## 2 METHODS

This study was carried out in accordance with the research ethics guidelines of Kenya's Ministry of Education, Science, and Technology. All animal use was in accordance with the provisions of the Prevention of Cruelty to Animals Act Cap 360 of the laws of Kenya, and the regulations established by the Kenya Veterinary Board.

### 2.1 Study site

We conducted the study at Mpala Research Centre (0°17N′, 36°52′ E; 1,800 m above sea level) in Laikipia County, Kenya. The study site receives 500–600 mm of rainfall annually in three rainy periods; April‐June (“long” rains), August (“continental” rains) and October‐November (“short” rains). The site is located in a black cotton soil (vertisol) wooded savanna ecosystem dominated by the whistling thorn tree (*Acacia drepanolobium*) and perennial grasses *Themeda triandra* Forssk., *Brachiaria lachnantha* (Hochst.) Stapf, and *Pennisetum stramineum* Peter. The site comprises a mosaic of low‐lying mounds build by termites belonging to the genus *Odontotermes* (Brody et al., 2010). These termite mounds can measure up to 10–20 m in diameter (Jouquet, Ranjard, Lepage, & Lata, 2005), and are regularly spaced (20–120 m between mounds; Pringle et al., 2010).

**Figure 1:**
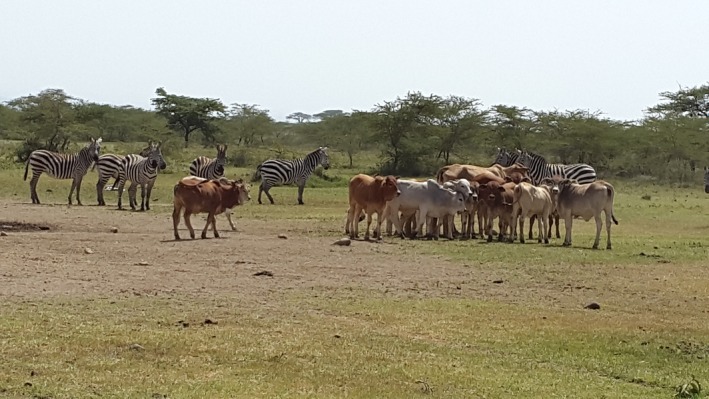
Boran cattle and plains zebras sharing a habitat in an African savanna ecosystem [Colour figure can be viewed at http://wileyonlinelibrary.com]

Mpala Research Centre is established on Mpala Conservancy, a 20,000‐ha property that integrates livestock production with wildlife conservation. The study site hosts livestock (mainly Boran cattle [*Bos indicus*; Figure 1]) and a largely intact community of large native wild herbivores. The most common wild herbivores in the study site include plains zebras (*Equus burchelli*; Figure 1), Grant's gazelle (*Gazella granti*), oryx (*Oryx beisa*), buffalo (*Syncerus caffer*), elephants (*Loxodonta africana*) and giraffes (*Giraffa camelopardalis*).

### 2.2 Study plots and attributes measured

This research was carried out in the Kenya Long‐term Exclosure Experiment (KLEE). The KLEE experimental setup comprises six herbivory treatments that exclude or allow different combinations of three groups of large herbivores (>15 kg), namely, cattle (“C”), medium‐sized wild ungulates (20–1,000 kg, “W”) and megaherbivores (elephants and giraffes, “M”). The treatments are applied to 4‐ha (200 m × 200 m) plots replicated across three experimental blocks (see Young, Okello, Kinyua, & Palmer, 1998 for details). For this study, we used the herbivory treatment plots that cattle accessed exclusively (C), and those they shared with medium‐sized wild ungulates in the absence (WC) or presence (MWC) of megaherbivores.

Normally, a herd of 100–120 head of cattle regularly accesses each of C, WC, and MWC treatment plots for 2 hr on each of two to three consecutive days, typically three to four times yearly depending on forage availability. However, for the purpose of our study, we replaced these regular cattle runs with small experimental heifer herds during the years 2007 and 2008. Each 4‐ha plot was accessed by four experimental heifers for 4 consecutive months, resulting in a stocking rate of approximately 0.3 cattle ha^−1^ year^−1^ .

We assessed several selected attributes of termite mounds, cattle foraging behavior, and herbaceous vegetation across study plots. Termite mound attributes measured were density (number of mounds per unit area), individual mound surface area, and total and percent area covered by mounds. Cattle attributes included percentage of bites taken on each patch type (on and off termite mound), patch type selection, diet species composition and selection. Vegetation attributes were aerial cover, leafiness and leaf greenness, and plant species composition on each patch type.

### 2.3 Assessment of termite mound attributes

We mapped mature termite mounds in May 2014 and again in July 2015. We conducted searches for termite mounds by walking adjacent 10 m‐wide transects covering the entire area of each 4‐ha plot. We used a Trimble Juno 3B GPS with meter‐level accuracy to map the outline of each termite mound. We delineated the edges of termite mounds using their topography and vegetation. *Odontotermes* mounds rise up to 0.5 m off the ground (Darlington & Bagine, 1999) and, when active, feature visible vents used for both nest ventilation and humidity control (Pomeroy, 2005). In addition to topographical features, these termite mounds are characteristically treeless but vegetated, and their edges can generally be delineated from background vegetation by a visible shift in plant community composition. Together, these features make most termite mounds visible from distances well over ten meters, although smaller mounds may be harder to identify until closer.

Termite mound data were imported into QGIS 1.8.0 (QGIS Development Team, 2012). We checked all GPS tracks around termite mounds for geometric validity and then calculated the area of all individual mounds. Individual mound data were summed within each plot to calculate total and percent termite mound area per plot.

### 2.4 Vegetation sampling

We measured herbaceous vegetation cover, leafiness and leaf greenness and species composition on and off termite mound patches in early April 2007. For termite mound patches, we randomly selected four distinct termite mounds (diameter >5 m) in each plot, and placed five 5‐m line transects (~1 m between transects) on each selected mound. For off‐mound patches, we subdivided each plot into eight 100 m × 50 m subplots, and placed a 25‐m line transect approximately centrally in each subplot, avoiding any areas covered or influenced by termite mounds. Both of these sampling methods are area‐independent, and therefore, not biased by the area surveyed, or its shape. Each was most appropriate for the landscape feature being sampled. A 25‐m transect surveys a broader subsample of the off‐mound landscape, but was not possible for the (more constrained) termite mounds, where the sampling was adjusted (but not in a way that would bias the estimates of the vegetation attributes measured).

We used the line point intercept method to estimate herbaceous vegetation attributes on each patch type. The procedure involved pacing each transect, dropping a 1‐m pin perpendicular to the ground every one pace (~1 m), and recording the first pin hit by species and plant part (green [live] leaf, brown [dead] leaf, green stem, and brown stem). Pins not intercepted by any vegetation were recorded as misses. For each plot, we calculated total aerial herbage cover both off and on termite mounds as the total number of pins intercepted by vegetation divided by the total number of pins dropped. We also calculated the relative aerial cover of each herbage species by dividing the total number of pin hits on that species by the total number of vegetation pin hits. In addition, we calculated herbage leafiness and leaf greenness as the number of pin hits on all leaves and green leaves divided by the total number of pin hits on all plant parts and on all leaves, respectively. These herbage quality attributes were also calculated for each of the three most common herbaceous species (the grasses *B. lachnantha*,* P. stramineum,* and *T. triandra*).

### 2.5 Cattle foraging observations

**Figure 2:**
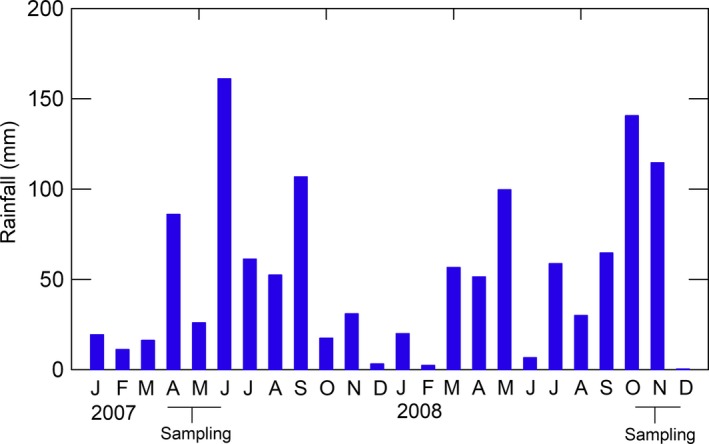
Monthly rainfall during the study and sampling periods for vegetation and cattle foraging attributes. Rainfall data were obtained from a gauging station located at Mpala Research Centre [Colour figure can be viewed at http://wileyonlinelibrary.com]

We conducted foraging observations during two sampling periods; early April to early June 2007 and late October to early December 2008. Both periods were generally relatively wet (Figure 2). For each sampling period, we used a separate set of 36 Boran test heifers (age 2–3.5 years, weight 261 kg ± 43 *SD*) allotted to the nine experimental plots (four heifers per plot). For the purpose of another study that was ongoing (Odadi et al., 2011), the test heifers had been foraging in their respective plots for 6–8 weeks before the start of each sampling period.

Individual test heifers were observed in four (April–June 2007) or five (October–December 2008) 5‐min focal periods once every 2 weeks. During each focal period, the number of bites taken on different species was recorded and bites categorized based on whether they were cropped on or off termite mounds. All observations and recordings were executed by three pairs of trained and experienced crews. For each bi‐weekly survey, the crew pairs were each assigned treatment plots within one of the three experimental blocks. Plots within each block were sampled on different days in a sequence randomly predetermined at the start of each bi‐weekly survey. All observations were made between 0900 and 1100 hr.

### 2.6 Determination of patch type and diet selection

For each plot, we pooled data from all sampling days per patch type per sampling period, and calculated the proportion of bites cattle took on each patch type, and plant species and functional type (forb and grass). We determined the degree of selection of patch types, and plant species and functional types using Jacobs’ (1974) index of selection *D*_*i*_ = (*p*_*i*_ − *c*_*i*_)/(*p*_*i*_ + *c*_*i*_ − *2p*_*i*_ *c*_*i*_), where *p*_*i*_ and *c*_*i*_ are the proportions of a given resource *i* in diet (bites) and in the environment (cover), respectively. The index ranges from −1 (total avoidance) through 0 (neutral selection) to 1 (total selection). Positive, neutral, and negative selection indices indicate the relative use of a resource is, respectively, higher than, equal to and lower than its relative availability.

### 2.7 Data analysis

Experimental design comprised three herbivory treatments (C [cattle only], WC [medium‐sized wild herbivores and cattle], and MWC [megaherbivores, medium‐sized wild herbivores, and cattle]) applied to 4‐ha plots and replicated across three experimental blocks. Each treatment plot consisted of two natural patch types (off vs. on termite mounds). Because measured attributes generally appeared similar between WC and MWC, we lumped these two treatments together for data analysis. We conducted univariate data analyses using linear mixed‐effects models. For termite mound attributes, we specified herbivory treatment (C vs. WC and MWC) and experimental blocks as fixed and random factors, respectively. For percentage bites on termite mounds, we included herbivory treatment as a fixed factor and experimental blocks and plots nested within blocks as random factors. Models for all other attributes included herbivory treatment, patch type, and herbivory treatment by patch type interaction as fixed factors, and blocks and plots nested within blocks as random factors. To determine whether individual forage species were significantly positively or negatively selected on each patch type, we performed one‐sample Student's *t‐*tests on selection indices with 0 as the hypothetical mean.

We used a permutational multivariate analysis of variance, PERMANOVA (Anderson, 2001), to test for potential differences in plant community composition and cattle bites across patch types, herbivory treatments, and their interaction. Permutations (*N* = 999) were constrained within experimental blocks to control for block effects. We used nonmetric multidimensional scaling (NMDS) to visualize compositional differences. For both PERMANOVA analysis and NMDS visualization, we calculated vegetation sample dissimilarity using Bray–Curtis distance measures.

All analyses were performed in R (R 3.3.0; R Core Team, 2016). Linear mixed‐effects models were run using the *nlme* package (Pinheiro, Bates, DebRoy, & Sarkar, 2016). We arcsine square root transformed all percentage data during analysis to meet normality and heteroscedasticity assumptions. For all other data, we visually inspected residual plots and applied appropriate transformations when necessary to meet model assumptions. Because model residuals for selection of patch types and plant functional types appeared heteroscedastic even after transformation, we incorporated the *varIdent* function into these models to allow for unequal variance across herbivory treatments. We performed Tukey's post hoc tests in the package *multcomp* (Hothorn, Bretz, & Westfall, 2008) to separate means for significant (*p *<* *0.05) or nearly significant (*p *<* *0.1) herbivory treatment by patch type interaction effects. All interactions with *p *>* *0.1 were dropped from the models. We report all data as untransformed means ± *SE*. R code, and statistical outputs are presented in Supporting Information Appendix S1.

## 3 RESULTS

### 3.1 Termite mound attributes and selection by cattle

**Figure 3:**
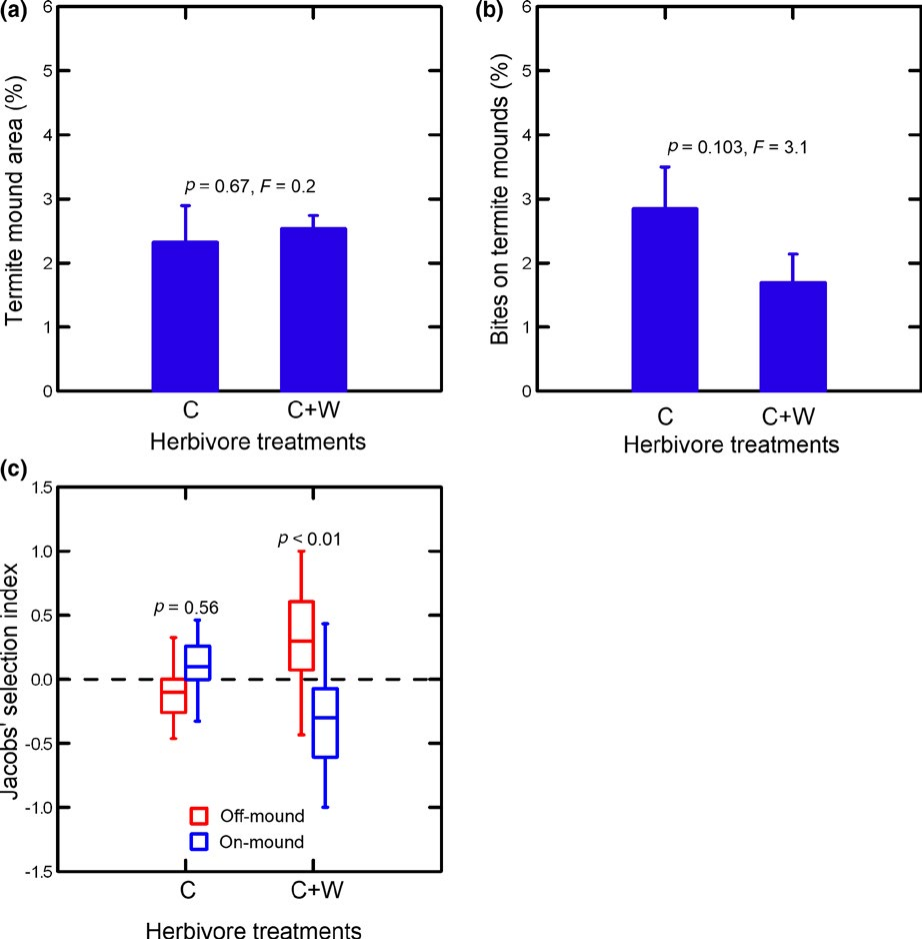
Percentage area covered by termite mounds (a), percentage of bites taken by cattle on termite mounds (b), and cattle selection (Jacobs’ indices) of patch types (c) in herbivory treatment plots cattle accessed exclusively (C) or shared with wild herbivores (C + W). Bar plot data are means with standard errors. Box plots show medians (lines), 25%–75% quartiles (boxes), and ranges (whiskers). For bar plots, *p*‐values compare herbivory treatments. For box plots, *p*‐values compare the two patch types (i.e., on vs. off termite mounds) within each herbivory treatment [Colour figure can be viewed at http://wileyonlinelibrary.com]

Overall, individual termite mounds averaged 8.3 ± 1.3 per hectare. Termite mounds covered 246.5 m^2^/ha ± 30.1, which translates to 2.5% ± 0.3 of the study site. The mean area covered by an individual mound was 32.8 m^2^ ± 4.5, which implies an approximate diameter of 6.4 m^2^ ± 0.4, assuming a circular shape. None of the measured termite mound attributes differed significantly between plots cattle accessed exclusively and those they shared with wild herbivores (density 7.8 mounds/ha ± 1.5 vs. 8.5 mounds/ha ± 1.9, *p *=* *0.79, *F *=* *0.1; total area 232 m^2^/ha ± 81 vs. 253 m^2^/ha ± 29 [see Figure 3a for corresponding percentages] *p *=* *0.67, *F* = 0.2; area per mound 28.4 m^2^ ± 4.4 vs. 35.0 m^2^ ± 6.5, *p *=* *0.53, *F *=* *0.5).

We counted a total of 281,327 bites of cattle in 1,376 (5‐min) focal periods. Overall, 2.1% ± 0.4 of these bites were taken on termite mounds. Cattle took 50% more bites on termite mounds in plots not shared with wild herbivores, although this difference was not quite statistically significant (*p *=* *0.103; Figure 3b). However, selection of termite mounds was strongly influenced by an interaction between herbivory treatment and patch type interaction (*p *=* *0.002, *F *=* *13.6; Figure 3c). In particular, selection index of termite mounds was lower in the shared plots than in plots cattle accessed exclusively (*p *=* *0.045). In addition, cattle used termite mounds in direct proportion to their availability (neutral selection) when they accessed foraging areas exclusively (*p *=* *0.56), but avoided (negatively selected) termite mounds when they shared foraging areas with wild herbivores (*p *<* *0.01).

### 3.2 Herbaceous vegetation attributes

Total herbage cover was influenced by an interaction between herbivory treatment and patch type (*p *<* *0.01, *F *=* *16.7; Table 1). In particular, herbage cover did not differ between patch types in plots that cattle did not share with wild herbivores whereas it was 16% lower (*p *<* *0.01) on termite mounds compared to off in the shared plots. In addition, total herbage cover on (but not off) termite mounds was 18% lower (*p *<* *0.01) in the shared plots than in plots cattle accessed exclusively. Overall, herbage was leafier off than on termite mounds (*p *=* *0.01, *F *=* *10.6; Table 1). On the other hand, however, herbage leaves were 50% greener on than off termite mounds (*p *<* *0.01, *F *=* *17.8; Table 1). Both overall herbage leafiness and leaf greenness did not differ between herbivory treatments (both *p *>* *0.81, *F *<* *0.1; Table 1). However, analysis of individual species showed that *B. lachnantha* was leafier in shared than unshared plots for off‐mound (*p *<* *0.01) but not for on‐mound (*p *=* *0.92) patches (herbivory by patch type interaction *p *=* *0.04, *F *=* *9.1; Table 1). In addition, this grass was leafier off than on termite mounds in shared (*p *=* *0.04) but not in unshared (*p *=* *0.27) plots. *Pennisetum stramineum* tended to be leafier off than on termite mounds (*p *=* *0.09, *F *=* *3.8), but its leaves were greener on termite mounds (*p *<* *0.01, *F *=* *15.2; Table 1). *Themeda triandria* was leafier, and its leaves greener, on than off termite mounds (both *p *=* *0.04, *F *>* *12.1; Table 1). In addition, this grass tended to be leafier in shared than unshared plots (*p *=* *0.07, *F *=* *5.1), although its leaf greenness did not differ between herbivory treatments (*p *=* *0.43, *F *=* *0.7). Both leafiness and leaf greenness of *P*.* stramineum* did not differ significantly between herbivory treatments (both *p *>* *0.61, *F *<* *0.3; Table 1).

**Table 1:** Attributes of overall herbaceous vegetation and three dominant species (mean [%] ± *SE*) off and on termite mounds in plots cattle accessed exclusively (C) or shared with wild herbivores (C + W)

Species and attributes	Patch type	Herbivory treatments		
		C	C + W	Overall
Overall herbage				
Cover	Off‐mound	96.2 ± 2.1	94.7 ± 1.1	95.2 ± 1.0
	On‐mound	96.0^a^ ± 2.0	79.2^b^ ± 2.8	84.7 ± 3.4
Leafiness	Off‐mound	62.2 ± 1.9	66.3 ± 1.8	64.9^A^ ± 1.5
	On‐mound	56.6 ± 4.9	54.3 ± 3.3	55.1^B^ ± 2.6
Leaf greenness	Off‐mound	33.4 ± 9.2	33.0 ± 2.9	33.1^A^ ± 3.3
	On‐mound	55.1 ± 6.1	48.9 ± 1.6	49.6^B^ ± 2.1
*Brachiaria lachnantha*				
Leafiness	Off‐mound	76.0^a^ ± 1.4	87.3^b(A)^ ± 1.1	83.5 ± 2.1
	On‐mound	82.9 ± 2.9	80.4^(B)^ ± 3.1	81.2 ± 2.2
Leaf greenness	Off‐mound	36.4 ± 6.3	36.7 ± 3.4	36.6^A^ ± 2.9
	On‐mound	56.3 ± 6.3	57.5 ± 2.5	57.1^B^ ± 2.3
*Themeda triandra*				
Leafiness	Off‐mound	52.2 ± 3.8	60.4 ± 3.1	57.7^A^ ± 2.7
	On‐mound	—	73.9 ± 3.9	72.1^B^ ± 3.3
Leaf greenness	Off‐mound	22.6 ± 8.9	28.7 ± 3.7	26.7^A^ ± 3.7
	On‐mound	—	47.2 ± 12.1	47.9^B^ ± 8.6
*Pennisetum stramineum*				
Leafiness	Off‐mound	59.2 ± 2.7	59.5 ± 3.9	59.6 ± 2.6
	On‐mound	55.6 ± 4.1	51.9 ± 3.3	53.1 ± 2.5
Leaf greenness	Off‐mound	29.3 ± 11.7	26.9 ± 4.1	27.7^A^ ± 4.3
	On‐mound	49.3 ± 4.7	46.5 ± 1.9	47.4^B^ ± 2.1

*Notes*

Blanks imply insufficient data.

For each attribute, column means with different uppercase superscripts differ significantly (*p *<* *0.05) between patch types (i.e., on vs. off termite mounds) whereas row means with different lowercase letters differ significantly between herbivory treatments.

**Figure 4:**
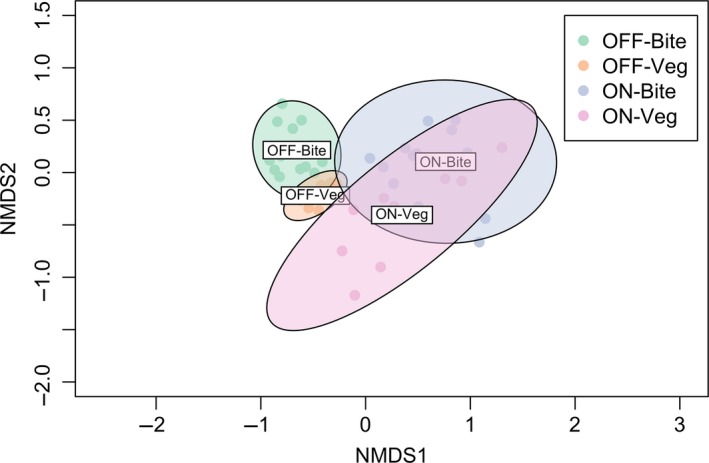
Nonmetric multidimensional scaling (NMDS) ordination plots showing differences in herbaceous vegetation species composition both in the environment and in cattle bites between termite mound and off‐mound areas [Colour figure can be viewed at http://wileyonlinelibrary.com]

Herbage cover both on and off termite mounds was dominated by grasses (relative cover ≥94%); forbs were far less abundant (Table 2). The overall relative cover of grasses (vs. forbs) was not significantly influenced by patch type or herbivory treatment (both *p *>* *0.68, *F *<* *0.2; Table 2). There was a significant difference between plant communities on and off termite mounds (PERMANOVA *F*_1,17_ = 98.45, *R* ^2^ = 0.85, *p *<* *0.001; Figure 4), but no significant difference between plant communities in different herbivore treatments (*F*_1,17_ = 1.85, *R* ^2^ = 0.02, *p *=* *0.14). Off termite mounds, herbage cover was dominated by the grasses *B. lachnantha*,* T. triandra* and *P. stramineum*; each comprised between 15% and 33% of total herbage cover on this patch type (Table 2). On termite mounds, herbage was predominantly comprised of *P. stramineum* (>85%), while *B. lachnantha*,* T. triandra* and *P. mezianum* were much less common (≤3% each). Other common species (relative cover >1%) were the grasses *Lintonia nutans* and *Bothriochloa insculpta*, and the forb *Pseudognaphalium* sp. Analyses of individual species showed that *P. stramineum* was more common on than off termite mounds, whereas all other major species (except *Pseudognaphalium* sp.) were more common off termite mounds (all *p *<* *0.01, *F *>* *23.9; Table 2). Individual species relative covers of did not differ significantly between herbivory treatments (all *p *>* *0.13, *F *<* *3.3; Table 2).

**Table 2:** Relative cover (mean [%] ± *SE*) of major herbage plants off and on termite mounds in plots cattle accessed exclusively (C) or shared with wild herbivores (C + W)

Species	Patch type	Herbivory treatments		
		C	C + W	Overall
*Brachiaria lachnantha*	Off‐mound	32.9 ± 3.5	26.5 ± 3.3	28.6^A^ ± 2.6
	On‐mound	2.7 ± 1.4	3.2 ± 1.1	3.0^B^ ± 0.8
*Pennisetum stramineum*	Off‐mound	29.4 ± 3.3	25.4 ± 2.4	26.7^A^ ± 1.9
	On‐mound	85.4 ± 7.4	86.9 ± 2.5	86.4^B^ ± 2.7
*Themeda triandra*	Off‐mound	14.9 ± 2.0	20.5 ± 2.4	18.6^A^ ± 1.9
	On‐mound	0.3 ± 0.3	2.2 ± 0.9	1.6^B^ ± 0.6
*Pennisetum mezianum*	Off‐mound	8.3 ± 2.1	10.7 ± 2.0	9.9^A^ ± 1.5
	On‐mound	0.0 ± 0.0	0.7 ± 0.4	0.5^B^ ± 0.3
*Lintonia nutans*	Off‐mound	5.5 ± 0.8	5.5 ± 0.5	5.5^A^ ± 0.4
	On‐mound	1.2 ± 1.2	1.0 ± 0.5	1.1^B^ ± 0.5
*Bothriochloa insculpta*	Off‐mound	3.6 ± 2.4	4.2 ± 1.5	4.0^A^ ± 1.2
	On‐mound	0.3 ± 0.3	0.2 ± 0.2	0.2^B^ ± 0.1
*Pseudognaphalium* spp.	Off‐mound	2.0 ± 0.7	1.7 ± 0.5	1.8 ± 0.4
	On‐mound	0.6 ± 0.6	3.1 ± 0.7	2.3 ± 0.7
Total grasses	Off‐mound	94.8 ± 1.6	94.5 ± 0.8	94.6 ± 0.7
	On‐mound	93.9 ± 3.2	94.3 ± 1.3	94.2 ± 0.3

*Notes*

Only species comprising mean relative cover >1% off or on termite mounds are included.

For each species, column means with different uppercase superscripts differ significantly (*p *<* *0.05) between patch types (i.e., on vs. off termite mounds).

### 3.3 Diet species composition and selection

Cattle principally consumed grasses both on and off termite mounds (>98% bites; Table 3). Cattle positively selected grasses (and avoided forbs, overall) on both patch types (on mound *p *=* *0.01, *t *=* *3.2; off mound *p *<* *0.01, *t *=* *9.6; Table 3). Cattle consumed and selected grasses more (and forbs less) on than off termite mounds (all *p *<* *0.02, *F *>* *7.2; Table 3). Both selection and relative consumption of grasses or forbs did not differ significantly between herbivory treatments (all *p *>* *0.18, *F *<* *2.4; Table 3). There was a significant difference between the plants cattle consumed on and off termite mounds (PERMANOVA *F*_1,35_ = 203.2, *R* ^2^ = 0.86, *p *<* *0.001; Figure 4), but no significant difference between plant communities in different herbivore treatments (*F*_1,35_ = 0.44, *R* ^2^ = 0.001, *p *=* *0.51). Off termite mounds, cattle mainly ate *B. lachnantha* (~60%), *T. triandra* (17%–22%) and *P*.* stramineum* (12%–19%) (Table 3). On termite mounds, however, they primarily ate *P*.* stramineum* (86%–87%), while eating *B. lachnantha* (6%–11%) and *T. triandra* (<5%) less frequently (Table 3). Cattle ate *P*.* mezianum*,* L. nutans* and *B. insculpta* much less frequently (≤2% both on and off mounds; Table 3). Cattle consumed a higher proportion of *P. stramineum* on than off termite mounds (*p *<* *0.01, *F *=* *183.8; Table 1). On the other hand, all other species comprised higher proportions of bites off than on termite mounds (*p *<* *0.01, *F *=* *23.0). Apart from *B. insculpta* which was consumed more in shared than unshared plots (*p *=* *0.02, *F *=* *6.4), consumption of all other species did not differ significantly between herbivore treatments (all *p *>* *0.41, *F *=* *0.7) (Table 3).

**Table 3:** Relative consumption and selection (Jacobs’ indices, *D*) of major forage plants by cattle off and on termite mounds in plots cattle accessed exclusively (C) or shared with wild herbivores (C + W)

Species	Patch type	Herbivory treatments					
		C		C + W		Overall	
		Bites (%)	*D*	Bites (%)	*D*	Bites (%)	*D*
*Brachiaria lachnantha*	Off‐mound	59.6 ± 3.2	0.41 ± 0.10	60.0 ± 1.8	0.61 ± 0.07	59.8^A^ ± 1.6	**0.54 **±** **0.06
	On‐mound	6.1 ± 5.0	0.44 ± 0.37	10.6 ± 4.6	0.74 ± 0.11	9.0^B^ ± 3.4	**0.66 **±** **0.13
*Themeda triandra*	Off‐mound	16.8 ± 3.0	−0.20 ± 0.14	21.9 ± 2.6	−0.19 ± 0.06	20.2^A^ ± 2.0	−**0.19 **±** **0.06
	On‐mound	4.7 ± 4.1	—	0.3 ± 0.2	−0.42 ± 0.37	1.9^B^ ± 1.5	−0.31 ± 0.33
*Pennisetum stramineum*	Off‐mound	18.6 ± 4.8	−0.03 ± 0.12	11.9 ± 2.2	−0.27 ± 0.10	14.1^A^ ± 2.2	−**0.19 **±** **0.08
	On‐mound	87.3 ± 6.0	−0.19 ± 0.48	85.8 ± 4.8	−0.06 ± 0.25	86.3^B^ ± 3.7	−0.10 ± 0.21
*P. mezianum*	Off‐mound	1.2 ± 0.3	−0.67 ± 0.03	1.2 ± 0.3	−0.73 ± 0.06	1.2^A^ ± 0.2	−**0.71 **±** **0.04
	On‐mound	0.2 ± 0.2	—	0.2 ± 0.1	−0.60 ± 0.40	0.2^B^ ± 0.1	−0.60 ± 0.40
*Lintonia nutans*	Off‐mound	1.6 ± 0.4	−0.48 ± 0.05	1.9 ± 0.3	−0.37 ± 0.03	1.8^A^ ± 0.2	−**0.40 **±** **0.03
	On‐mound	0.7 ± 0.4	—	0.5 ± 0.3	−0.19 ± 0.49	0.6^B^ ± 0.2	−0.36 ± 0.41
*Bothriochloa insculpta*	Off‐mound	0.5 ± 0.2	−0.62 ± 0.14	2.0 ± 0.6	−0.04 ± 0.24	1.5^A^ ± 0.4	−0.24 ± 0.18
	On‐mound	0.0 ± 0.0	—	0.1 ± 0.1	0.33 ± 0.67	0.1^B^ ± 0.1	0.00 ± 0.58
Total grasses	Off‐mound	99.0 ± 0.3	0.47 ± 0.15	99.3 ± 0.1	0.68 ± 0.05	99.2^A^ ± 0.1	**0.61 **±** **0.06
	On‐mound	99.1 ± 0.5	0.20 ± 0.60	99.6 ± 0.2	0.90 ± 0.05	99.4^B^ ± 0.2	**0.67 **±** **0.21

*Notes*

Data are means ± *SE*. Only species comprising an average of >1% of total bites either on or off mounds are included. Overall mean selection indices listed in bold differ significantly from 0 (neutral selection). Blanks imply insufficient data.

For each species, column means with different superscripts differ significantly (*p *<* *0.05) between patch types (i.e., on vs. off termite mounds).

Among the major species comprising cattle diet, *B. lachnantha* was the most preferred species, and was significantly positively selected both on and off termite mounds (on *p *<* *0.01, *t *=* *5.2; off *p *<* *0.01, *t *=* *8.9; Table 3). However, all other major species (except *B. insculpta*) were negatively selected off mounds (all *p *≤* *0.06, *t *≤* *−2.2), but neutrally selected on mounds (all *p *≥* *0.27, −1.5 *<* *t *<* *−0.5). *Bothriochloa insculpta* was neutrally selected on both patch types (on *p *>* *0.99, *t *=* *0.0, off *p *=* *0.24, *t *≤* *−1.3). Selection index of *B. lachnantha* tended to be higher in shared than unshared plots (*p *=* *0.07, *F *=* *5.2; Table 3). However, selection indices of all the other species did not differ between herbivory treatments (all *p *>* *0.12, *F *<* *3.6; Table 3).

## 4 DISCUSSION

In this study, we assessed for the first time the effects of shared foraging with wild herbivores on the extent to which cattle select termite mounds as foraging patches in an African savanna ecosystem. We found that termite mound selection index was lower in foraging areas cattle shared with wild herbivores than in foraging areas cattle accessed exclusively. Furthermore, cattle used termite mounds in direct proportion to their relative availability (neutral selection) when they were the only herbivores present, but used them less than their availability (negative selection) when they shared foraging areas with wild herbivores. These findings support our hypothesis that shared foraging with wild herbivores diminishes the degree of selection of termite mound patches by cattle.

The observed differences in the magnitude and direction of selection of termite mounds by cattle between herbivory treatments were related to differences in termite mound herbage cover and off‐mound forage leafiness between these treatments. In particular, reduced termite mound selection in foraging areas cattle shared with wild herbivores was partly related to lower herbage cover on termite mounds in these shared areas than in areas cattle accessed exclusively. In addition, the shift in termite mound selection from neutral selection in areas cattle accessed exclusively to negative selection in areas they shared with wild herbivores was related to lower herbage cover on than off termite mounds in the shared areas. These herbage cover differences indicate differences in herbage quantity because herbage cover correlates positively with biomass in this ecosystem (Veblen, Porensky, Riginos, & Young, 2016). Our results show that when herbage cover is similar between mounds and off‐mound areas, as was the case in unshared herbivory treatment plots, cattle use termite mounds in equal proportion to their availability. However, cattle avoid (negatively select) termite mounds when herbage cover is lower on than off termite mounds, as was seen in the shared plots. We suggest that there is a threshold of herbage cover below which cattle negatively select termite mounds despite enhanced herbage leaf greenness; in other words, cattle trade off quality for quantity. In an alternative manner, there is a threshold of herbage cover reduction on termite mound versus off‐mound patches above which cattle negatively select termite mound patches. In other words, the extent to which cattle select termite mounds depends on the level of herbage cover on mounds relative to herbage cover in the surrounding matrix. While these thresholds are unclear from this study, the observed altered level and direction of termite mound selection in foraging areas cattle shared with wild herbivores suggest that the thresholds were exceeded in these shared areas.

It appears that the relative use of termite mounds by herbivores depends on the nutritional differences between mounds and off‐mound areas; larger differences result in greater relative use of termite mounds (Davies, Levick, et al., 2016). Therefore, the observed difference in cattle selection of termite mounds between herbivory treatments was also partly attributable to the observed higher off‐mound leafiness of *B. lachnantha* (the principal cattle diet species) in plots cattle shared with wild herbivores than in plots cattle accessed exclusively. We posit that higher off‐mound leafiness of this grass in shared than unshared foraging areas reduced the nutrient content differences between on and off termite mound patches in the shared areas, thereby diluting the degree of termite mound selection by cattle when foraging in the shared areas. Whereas the relative consumption of *B. lachnantha* did not differ significantly between herbivory treatments, it is notable that its selection index tended to be higher in the shared plots, perhaps as a result of its increased leafiness.

While we did not perform measurements during the dry season, we postulate that competition for termite mounds between cattle and wild herbivores would be more pronounced during dry periods. This is because during dry periods, forage quality declines, and herbivores are likely to be under increased pressure to locate forage resources with adequate nutrient concentrations (Owen‐Smith & Novellie, 1982). As opposed to vegetation growing off termite mounds, vegetation on mounds maintains high levels of essential nutrients even during the dry season (Grant & Scholes, 2006; Naiman et al., 2003). Therefore, large herbivores are likely to rely more heavily on termite mounds and similar nutrient‐rich hotspots (e.g., glades and burned areas) during dry periods, as has also been reported in our study ecosystem (Odadi et al., 2017; Veblen, 2012) and elsewhere (Davies, Levick, et al., 2016; Davies, van Rensburg, et al., 2016). Increased nutritional importance of mounds to herbivores during the dry season could magnify the negative effects of shared foraging with wild herbivores on selection of termite mounds by cattle.

That altered selection of termite mounds by cattle when they shared foraging areas with wild herbivores was unlikely to harm cattle during the wet season is supported by previous findings in this ecosystem. A parallel study conducted using the same experimental plots and heifers showed that wild herbivores facilitated cattle through enhanced forage and diet quality during the wet season, but competed with them through reduced forage availability during the dry season (Odadi et al., 2011). Because the present study was conducted during the wet season, facilitation would not have occurred (in the concurrent study) if the demonstrated wildlife‐driven reduced termite mound selection by cattle was nutritionally detrimental to cattle. The fact that facilitation still occurred suggests that any nutritional effects of altered selection of termite mounds were not sufficient to overturn the overall pattern of interaction between cattle and wild herbivores during the wet season. By contrast, reduced selection of termite mounds could be detrimental to cattle during the dry season when both forage quality and quantity decline. Parallel to these postulated season‐dependent effects, shared foraging with wild herbivores in burned areas, which like termite mounds are also nutrient‐rich hotspots, was nutritionally detrimental to cattle during dry season but not during wet season (Odadi et al., 2017). Burned areas help cattle meet their nutritional (crude protein and digestible dry matter intake) requirements for maintenance and growth, but intense competition for these nutrient hotspots with wild herbivores during the dry season impairs the ability of cattle to meet these requirements (Odadi et al., 2017). While we did not assess the actual nutritional implications of the effects of reduced termite mound selection on cattle, we suspect that they could be generally similar to those for burned areas.

Cattle diet species composition and selection differed between termite mounds and off‐mound areas because termite mounds harbored compositionally different herbaceous vegetation compared with the off‐mound areas. Such compositional differences, which have been reported in other savanna systems (Davies et al., 2014; Muvengwi et al., 2017), are normally associated with altered soil properties such as texture, moisture content and nutrient status on termite mounds (Evans et al., 2011; Meyer et al., 1999; Sileshi et al., 2010). The higher overall grass selection, and selection of most individual forage species, on than off termite mounds are consistent with previous findings elsewhere showing positive effects of termite mounds on selection of individual plant species by large herbivores (Muvengwi et al., 2014). These differences appear to be related to the observed higher leaf greenness on termite mounds, which indicates higher forage nutritional quality (Wang, Wang, Shi, & Omasa, 2014). Forage quality enrichment through increased foliar nitrogen and phosphorus on termite mounds has been previously reported in our study site (Brody et al., 2010; Fox‐Dobbs et al., 2010). We posit that the observed higher leaf greenness on mounds than off‐mound patches is related to soil differences between these patch types. While we did not assess soil differences between mounds and off‐mound patches in the present study, a previous assessment in our study site showed that soil phosphorus and nitrogen contents were 70% and 84% greater, respectively, for mounds than the surrounding matrix (Brody et al., 2010). Likewise, consistent with our findings, the positive effects of termite mounds on selection of individual plant species by large herbivores have also been reported elsewhere (Muvengwi et al., 2014). Our study shows that while cattle consume and select forage species differently between termite mounds and off‐mound areas, these patterns are not altered when cattle share habitat with wild herbivores. Therefore, the effects of shared foraging with wild herbivores on termite mound selection by cattle observed here appear to have occurred at foraging patch scale rather than at finer spatial scales.

The differences in herbage cover, forage leafiness and termite mound selection between shared and unshared plots are primarily associated with herbivory treatments because all the measured termite mound attributes were similar among plots. In other words, these effects were associated with recent herbivory rather than a legacy of herbivory treatments changing termite mound properties. Although we mapped termite mounds 7–8 years after foraging behavior surveys, this was not expected to bias our results because the presence of wild ungulates does not significantly influence the density and area covered by mature termite mounds in this landscape (G. K. Charles, unpublished data), which appear to be stable. Therefore, we have no reason to believe that any changes in termite mound properties over time would not be similar across our study plots. We associate the effects reported here to grazing activity of medium‐sized wild ungulates, and especially plains zebras which are by far the most common wild mammalian herbivores in the study site (Odadi et al., 2017). Zebras are highly attracted to termite mounds in our study site (Brody et al., 2010), and are thus likely to have the greatest impact on termite mounds. Other wild ungulates that frequent the study site and therefore possibly contributed to these effects are buffalos, oryx, elands, and Grant's gazelles (Odadi et al., 2017).

## 5 CONCLUSIONS

Our findings provide important insights into how cattle and wild herbivores interact on nutrient‐rich termite mounds when they share habitats in African savannas. The extent to which cattle select termite mounds as foraging patches reduces when they share foraging areas with wild herbivores partly because of reduced forage availability on termite mounds in these areas. Therefore, wild herbivores appear to compete with cattle for forage on termite mounds in these savannas. However, such competition seems to be of limited nutritional consequence to cattle during the wet season when they benefit from improved forage leafiness off termite mounds in areas they share with wild herbivores. While our study was conducted during wet periods, we posit that competition for termite mounds could intensify between these herbivore guilds during drier periods when the nutritional importance of termite mounds to both herbivores is likely to increase. It would be worth investigating the actual nutritional consequences of competition for termite mound forage on cattle and wild herbivores, and the patterns of variation of such effects across time, herbivore body sizes and feeding habits. In the meantime, we recommend application of grazing management practices that could minimize the effects of such competition (e.g., reducing stock numbers during dry periods) for better management of domestic and wild herbivore guilds and their shared habitats.

## CONFLICT OF INTEREST

None declared.

## AUTHOR CONTRIBUTIONS

WOO designed and executed cattle and vegetation surveys, performed data analyses (LMMs and Student's t tests), and wrote the manuscript; GKC designed and executed termite mound attributes measurements and performed NMDS and PERMANOVA analyses; TPY designed and maintained KLEE experimental plots, and provided advisory and logistical support; All authors read, critically revised, and approved the manuscript.

## DATA ACCESSIBILITY

Data are archived in figshare, https://doi.org/10.6084/m9.figshare.6848324.
