# Supplementary material for: Cattle select African savanna termite mound patches less when sharing habitat with wild herbivores
Source: Ecol Evol. 2018 Aug 19;8(17):9074–85. doi: 10.1002/ece3.4452 (PMC6157688; doi:10.1002/ece3.4452)
Supplement: Supplementary file 1 [file ECE3-8-9074-s001.pdf]

Appendix S1. R scripts and statistical results;

```
#Linear mixed effects models

km0708w=read.csv(file.choose())
require(nlme)
require(multcomp)

#Percent area covered by mounds
km0708wTMD07 <- subset(km0708w, YEAR=="2007" & LOCATION=="TMD",select=YEAR:TVL2S)
PAREA=lme(asin(sqrt(PAREA))~COMB,data = km0708wTMD07,random=~1|BLOCK,
+control=lmeControl(opt="optim"),na.action=na.omit)
anova(PAREA)
            numDF denDF  F-value p-value
(Intercept)     1     5 221.9570  <.0001
COMB            1     5   0.2096  0.6663

#Number of mounds
km0708wTMD07 <- subset(km0708w, YEAR=="2007" & LOCATION=="TMD",select=YEAR:TVL2S)
TMD_NUM=lme(TMD_NUM_PHA~COMB,data = km0708wTMD07,random=~1|BLOCK,
+control=lmeControl(opt="optim"),na.action=na.omit)
anova(TMD_NUM)
            numDF denDF  F-value p-value
(Intercept)     1     5 31.75014  0.0024
COMB            1     5  0.07900  0.7899

#Mean area per mound (total mound area divided by number of mounds)
km0708wTMD07 <- subset(km0708w, YEAR=="2007" & LOCATION=="TMD",select=YEAR:TVL2S)
AREA_PTMD=lme(AREA_PTMD~COMB,data = km0708wTMD07,random=~1|BLOCK,
+control=lmeControl(opt="optim"),na.action=na.omit)
anova(AREA_PTMD)
            numDF denDF  F-value p-value
(Intercept)     1     5 48.66659  0.0009
COMB            1     5  0.45041  0.5319

#Percentage bites on mounds
km0708wTMD <- subset(km0708w, LOCATION=="TMD",select=YEAR:TW_RB)
PBITES=lme(asin(sqrt(PBITES_OVRL))~COMB*YEAR,data = km0708wTMD,random=~1|BLOCK/PLOT,
+control=lmeControl(opt="optim"),na.action=na.omit)
anova(PBITES)
            numDF denDF   F-value p-value
(Intercept)     1    12 100.17705  <.0001
COMB            1    12   2.90605  0.1140
YEAR            1    12   1.62365  0.2267
COMB:YEAR       1    12   0.25245  0.6244

##Dropping the interaction term:
PBITES=lme(asin(sqrt(PBITES_OVRL))~COMB+YEAR,data = km0708wTMD,random=~1|BLOCK/PLOT,
+control=lmeControl(opt="optim"),na.action=na.omit)
anova(PBITES)
            numDF denDF   F-value p-value
(Intercept)     1    13 101.48660  <.0001
COMB            1    13   3.07687  0.1029
YEAR            1    13   1.71909  0.2125

#Selection of termite mounds (Based on Jacobs' Selection Index)
BPREFJ=lme(BPREFJ_OVRL~COMB*LOCATION,data = km0708w,random=~1|BLOCK/PLOT,
+control=lmeControl(opt="optim"),weights = varIdent(form =~ 1 | COMB),na.action=na.omit)
anova(BPREFJ)
              numDF denDF   F-value p-value
(Intercept)       1    16  0.000000  1.0000
COMB              1    14  0.000000  1.0000
LOCATION          1    16  2.640857  0.1237
COMB:LOCATION     1    16 13.598806  0.0020

##Post hoc test
km0708w$HL=interaction(km0708w$COMB,km0708w$LOCATION)
HL_BPREFJ=lme(BPREFJ_OVRL~-1+HL,data = km0708w,random=~1|BLOCK/PLOT,
+control=lmeControl(opt="optim"),weights = varIdent(form =~ 1 | COMB),na.action=na.omit)
summary(glht(HL_BPREFJ, linfct = mcp(HL = "Tukey", interaction_average=TRUE)))
```

```
         Simultaneous Tests for General Linear Hypotheses

Multiple Comparisons of Means: Tukey Contrasts


Fit: lme.formula(fixed = BPREFJ_OVRL ~ -1 + HL, data = km0708w, random = ~1 |
    BLOCK/PLOT, weights = varIdent(form = ~1 | COMB), na.action = na.omit,
    control = lmeControl(opt = "optim"))

Linear Hypotheses:
                       Estimate Std. Error z value Pr(>|z|)
C+W.OFF - C.OFF == 0     0.4143     0.1589   2.608   0.0451 *
C.TMD - C.OFF == 0       0.1989     0.1523   1.306   0.5592
C+W.TMD - C.OFF == 0    -0.2154     0.1589  -1.356   0.5270
C.TMD - C+W.OFF == 0    -0.2154     0.1589  -1.356   0.5270
C+W.TMD - C+W.OFF == 0  -0.6298     0.1652  -3.813   <0.001 ***
C+W.TMD - C.TMD == 0    -0.4143     0.1589  -2.608   0.0450 *
---
Signif. codes:  0 '***' 0.001 '**' 0.01 '*' 0.05 '.' 0.1 ' ' 1
(Adjusted p values reported -- single-step method)

#Total herbaceous vegetation cover
PCVEG=lme(asin(sqrt(PCVEG/100))~COMB*LOCATION,data = km0708w,random=~1|BLOCK/PLOT,
+control=lmeControl(returnObject=TRUE),na.action=na.omit)
anova(PCVEG)
              numDF denDF   F-value p-value
(Intercept)       1     7 1196.7930  <.0001
COMB              1     5   31.8016  0.0024
LOCATION          1     7   37.0511  0.0005
COMB:LOCATION     1     7   16.7097  0.0046

##Post-hoc test
km0708w$HL=interaction(km0708w$COMB,km0708w$LOCATION)
HL_PCVEG=lme(asin(sqrt(PCVEG/100))~-1+HL,data = km0708w,random=~1|BLOCK/PLOT,
+control=lmeControl(returnObject=TRUE),na.action=na.omit)
summary(glht(HL_PCVEG, linfct = mcp(HL = "Tukey", interaction_average=TRUE)))

         Simultaneous Tests for General Linear Hypotheses

Multiple Comparisons of Means: Tukey Contrasts


Fit: lme.formula(fixed = asin(sqrt(PCVEG/100)) ~ -1 + HL, data = km0708w,
    random = ~1 | BLOCK/PLOT, na.action = na.omit, control = lmeControl(returnObject = TRUE))

Linear Hypotheses:
                        Estimate Std. Error z value Pr(>|z|)
C+W.OFF - C.OFF == 0   -0.044540   0.040598  -1.097    0.688
C.TMD - C.OFF == 0     -0.008282   0.046878  -0.177    0.998
C+W.TMD - C.OFF == 0   -0.287517   0.040598  -7.082   <1e-04 ***
C.TMD - C+W.OFF == 0    0.036258   0.040598   0.893    0.806
C+W.TMD - C+W.OFF == 0 -0.242977   0.033148  -7.330   <1e-04 ***
C+W.TMD - C.TMD == 0   -0.279235   0.040598  -6.878   <1e-04 ***
---
Signif. codes:  0 '***' 0.001 '**' 0.01 '*' 0.05 '.' 0.1 ' ' 1
(Adjusted p values reported -- single-step method)

# Total herbaceous vegetation percentage leafinness
TVPCL=lme(asin(sqrt(TVPCL/100))~COMB*LOCATION,data = km0708w,random=~1|BLOCK/PLOT,
+control=lmeControl(returnObject=TRUE),na.action=na.omit)
anova(TVPCL)
              numDF denDF   F-value p-value
(Intercept)       1     7 2837.7866  <.0001
COMB              1     5    0.0913  0.7747
LOCATION          1     7   10.6952  0.0137
COMB:LOCATION     1     7    1.0774  0.3338

##Dropping the interaction term:
TVPCL=lme(asin(sqrt(TVPCL/100))~COMB+LOCATION,data = km0708w,random=~1|BLOCK/PLOT,
```

```
+control=lmeControl(returnObject=TRUE),na.action=na.omit)
anova(TVPCL)
            numDF denDF   F-value p-value
(Intercept)     1     8 2837.7957  <.0001
COMB            1     5    0.0908  0.7753
LOCATION        1     8   10.6319  0.0115

#Total herbaceous vegetation perentage leaf greenness
TVPCLG=lme(asin(sqrt(TVPCLG/100))~COMB*LOCATION,data = km0708w,random=~1|BLOCK/PLOT,
+control=lmeControl(returnObject=TRUE),na.action=na.omit)
anova(TVPCLG)
              numDF denDF  F-value p-value
(Intercept)       1     7 716.8270  <.0001
COMB              1     5   0.0562  0.8220
LOCATION          1     7  16.4831  0.0048
COMB:LOCATION     1     7   0.0695  0.7996

##Dropping the interaction term:
TVPCLG=lme(asin(sqrt(TVPCLG/100))~COMB+LOCATION,data = km0708w,random=~1|BLOCK/PLOT,
+control=lmeControl(returnObject=TRUE),na.action=na.omit)
anova(TVPCLG)
            numDF denDF  F-value p-value
(Intercept)     1     8 716.8246  <.0001
COMB            1     5   0.0605  0.8154
LOCATION        1     8  17.7538  0.0029

# B. lachnantha percentage leafinness
BLPCL=lme(asin(sqrt(BLPCL/100))~COMB*LOCATION,data = km0708w,random=~1|BLOCK/PLOT,
+control=lmeControl(returnObject=TRUE),na.action=na.omit)
anova(BLPCL)
              numDF denDF  F-value p-value
(Intercept)       1     5 7005.237  <.0001
COMB              1     5    7.151  0.0441
LOCATION          1     4    1.345  0.3106
COMB:LOCATION     1     4    9.062  0.0395

##Post-hoc test
km0708w$HL=interaction(km0708w$COMB,km0708w$LOCATION)
HL_BLPCL=lme(asin(sqrt(BLPCL/100))~-1+HL,data = km0708w,random=~1|BLOCK/PLOT,
+control=lmeControl(returnObject=TRUE),na.action=na.omit)
summary(glht(HL_BLPCL, linfct = mcp(HL = "Tukey", interaction_average=TRUE)))

         Simultaneous Tests for General Linear Hypotheses

Multiple Comparisons of Means: Tukey Contrasts


Fit: lme.formula(fixed = asin(sqrt(BLPCL/100)) ~ -1 + HL, data = km0708w,
    random = ~1 | BLOCK/PLOT, na.action = na.omit, control = lmeControl(returnObject = TRUE))

Linear Hypotheses:
                       Estimate Std. Error z value Pr(>|z|)
C+W.OFF - C.OFF == 0    0.14893    0.03747   3.975   <0.001 ***
C.TMD - C.OFF == 0      0.08649    0.04837   1.788   0.2749
C+W.TMD - C.OFF == 0    0.05710    0.04047   1.411   0.4877
C.TMD - C+W.OFF == 0   -0.06244    0.04326  -1.443   0.4676
C+W.TMD - C+W.OFF == 0 -0.09183    0.03420  -2.685   0.0358 *
C+W.TMD - C.TMD == 0   -0.02939    0.04589  -0.641   0.9175
---
Signif. codes:  0 ‘***’ 0.001 ‘**’ 0.01 ‘*’ 0.05 ‘.’ 0.1 ‘ ’ 1
(Adjusted p values reported -- single-step method)

# B. lachnantha percentage leaf greenness
BLPCLG=lme(asin(sqrt(BLPCLG/100))~COMB*LOCATION,data = km0708w,random=~1|BLOCK/PLOT,
+control=lmeControl(returnObject=TRUE),na.action=na.omit)
anova(BLPCLG)
              numDF denDF  F-value p-value
(Intercept)       1     5 584.3676  <.0001
COMB              1     5   0.0077  0.9335
LOCATION          1     4  26.7876  0.0066
```

```
COMB:LOCATION     1     4   0.0016  0.9696

##Dropping the interaction term:
BLPCLG=lme(asin(sqrt(BLPCLG/100))~COMB+LOCATION,data = km0708w,random=~1|BLOCK/PLOT,
+control=lmeControl(returnObject=TRUE),na.action=na.omit)
anova(BLPCLG)
            numDF denDF  F-value p-value
(Intercept)     1     5 582.4718  <.0001
COMB            1     5   0.0072  0.9356
LOCATION        1     5  29.9106  0.0028

# T. triandra percentage leafinness
TTPCL=lme(asin(sqrt(TTPCL/100))~COMB*LOCATION,data = km0708w,random=~1|BLOCK/PLOT,
+control=lmeControl(returnObject=TRUE),na.action=na.omit)
anova(TTPCL)
              numDF denDF   F-value p-value
(Intercept)       1     5 1912.3766  <.0001
COMB              1     5    4.4643  0.0883
LOCATION          1     2   10.3925  0.0842
COMB:LOCATION     1     2    0.0007  0.9817

##Dropping the interaction term:
TTPCL=lme(asin(sqrt(TTPCL/100))~COMB+LOCATION,data = km0708w,random=~1|BLOCK/PLOT,
+control=lmeControl(returnObject=TRUE),na.action=na.omit)
anova(TTPCL)
            numDF denDF   F-value p-value
(Intercept)     1     5 1832.3740  <.0001
COMB            1     5    5.1407  0.0727
LOCATION        1     3   12.1458  0.0399

# T. triandra percentage leaf greenness
TTPCLG=lme(asin(sqrt(TTPCLG/100))~COMB*LOCATION,data = km0708w,random=~1|BLOCK/PLOT,
+control=lmeControl(returnObject=TRUE),na.action=na.omit)
anova(TTPCLG)
              numDF denDF  F-value p-value
(Intercept)       1     5 78.94054  0.0003
COMB              1     5  0.75833  0.4237
LOCATION          1     2 13.70578  0.0658
COMB:LOCATION     1     2  1.01018  0.4207

##Dropping the interaction term:
TTPCLG=lme(asin(sqrt(TTPCLG/100))~COMB+LOCATION,data = km0708w,random=~1|BLOCK/PLOT,
+control=lmeControl(returnObject=TRUE),na.action=na.omit)
anova(TTPCLG)
            numDF denDF  F-value p-value
(Intercept)     1     5 87.64073  0.0002
COMB            1     5  0.74595  0.4272
LOCATION        1     3 13.19820  0.0359

# P. stramineum percentage leafinness
PSPCL=lme(asin(sqrt(PSPCL/100))~COMB*LOCATION,data = km0708w,random=~1|BLOCK/PLOT,
+control=lmeControl(returnObject=TRUE),na.action=na.omit)
anova(PSPCL)
              numDF denDF  F-value p-value
(Intercept)       1     7 772.2640  <.0001
COMB              1     5   0.2642  0.6292
LOCATION          1     7   3.6197  0.0988
COMB:LOCATION     1     7   0.2440  0.6365

##Dropping the interaction term:
PSPCL=lme(asin(sqrt(PSPCL/100))~COMB+LOCATION,data = km0708w,random=~1|BLOCK/PLOT,
+control=lmeControl(returnObject=TRUE),na.action=na.omit)
anova(PSPCL)
            numDF denDF  F-value p-value
(Intercept)     1     8 772.2640  <.0001
COMB            1     5   0.2805  0.6191
LOCATION        1     8   3.8432  0.0856

# P. stramineum percentage leaf greenness
PSPCLG=lme(asin(sqrt(PSPCLG/100))~COMB*LOCATION,data = km0708w,random=~1|BLOCK/PLOT,
```

```
+control=lmeControl(returnObject=TRUE),na.action=na.omit)
anova(PSPCLG)
              numDF denDF  F-value p-value
(Intercept)       1     7 374.3110  <.0001
COMB              1     5   0.0997  0.7650
LOCATION          1     7  14.0627  0.0072
COMB:LOCATION     1     7   0.0218  0.8868

##Dropping the interaction term:
PSPCLG=lme(asin(sqrt(PSPCLG/100))~COMB+LOCATION,data = km0708w,random=~1|BLOCK/PLOT,
+control=lmeControl(returnObject=TRUE),na.action=na.omit)
anova(PSPCLG)
            numDF denDF  F-value p-value
(Intercept)     1     8 374.3144  <.0001
COMB            1     5   0.1078  0.7560
LOCATION        1     8  15.2069  0.0045

# Relative cover of grasses
TGRC=lme(asin(sqrt(TGRC/100))~COMB*LOCATION,data = km0708w,random=~1|BLOCK/PLOT,
+control=lmeControl(returnObject=TRUE),na.action=na.omit)
anova(TGRC)
              numDF denDF  F-value p-value
(Intercept)       1     7 4286.890  <.0001
COMB              1     5    0.174  0.6936
LOCATION          1     7    0.036  0.8542
COMB:LOCATION     1     7    0.061  0.8127

##Dropping the interaction term:
TGRC=lme(asin(sqrt(TGRC/100))~COMB+LOCATION,data = km0708w,random=~1|BLOCK/PLOT,
+control=lmeControl(returnObject=TRUE),na.action=na.omit)
anova(TGRC)
            numDF denDF  F-value p-value
(Intercept)     1     8 4573.309  <.0001
COMB            1     5    0.186  0.6842
LOCATION        1     8    0.039  0.8488

# Relative cover B. lachnantha
BLRC=lme(asin(sqrt(BLRC/100))~COMB*LOCATION,data = km0708w,random=~1|BLOCK/PLOT,
+control=lmeControl(returnObject=TRUE),na.action=na.omit)
anova(BLRC)
              numDF denDF   F-value p-value
(Intercept)       1     7 191.44411  <.0001
COMB              1     5   0.33466  0.5880
LOCATION          1     7  87.67943  <.0001
COMB:LOCATION     1     7   0.77026  0.4092

##Dropping the interaction term:
BLRC=lme(asin(sqrt(BLRC/100))~COMB+LOCATION,data = km0708w,random=~1|BLOCK/PLOT,
+control=lmeControl(returnObject=TRUE),na.action=na.omit)
anova(BLRC)
            numDF denDF   F-value p-value
(Intercept)     1     8 191.44401  <.0001
COMB            1     5   0.33466   0.588
LOCATION        1     8  90.27189  <.0001

# Relative cover T. triandra
TTRC=lme(asin(sqrt(TTRC/100))~COMB*LOCATION,data = km0708w,random=~1|BLOCK/PLOT,
+control=lmeControl(returnObject=TRUE),na.action=na.omit)
anova(TTRC)
              numDF denDF  F-value p-value
(Intercept)       1     7  64.6026  0.0001
COMB              1     5   3.2089  0.1332
LOCATION          1     7 351.8845  <.0001
COMB:LOCATION     1     7   0.1540  0.7064

##Dropping the interaction term:
TTRC=lme(asin(sqrt(TTRC/100))~COMB+LOCATION,data = km0708w,random=~1|BLOCK/PLOT,
+control=lmeControl(returnObject=TRUE),na.action=na.omit)
anova(TTRC)
            numDF denDF  F-value p-value
```

```
(Intercept)     1     8  64.6026  <.0001
COMB            1     5   3.2089  0.1332
LOCATION        1     8 393.4987  <.0001

# Relative cover P. stramineum
PSRC=lme(asin(sqrt(PSRC/100))~COMB*LOCATION,data = km0708w,random=~1|BLOCK/PLOT,
+control=lmeControl(returnObject=TRUE),na.action=na.omit)
anova(PSRC)
              numDF denDF  F-value p-value
(Intercept)       1     7 572.6849  <.0001
COMB              1     5   0.4353  0.5386
LOCATION          1     7 133.5887  <.0001
COMB:LOCATION     1     7   0.0056  0.9427

##Dropping the interaction term:
PSRC=lme(asin(sqrt(PSRC/100))~COMB+LOCATION,data = km0708w,random=~1|BLOCK/PLOT,
+control=lmeControl(returnObject=TRUE),na.action=na.omit)
anova(PSRC)
            numDF denDF  F-value p-value
(Intercept)     1     8 572.6856  <.0001
COMB            1     5   0.4713  0.5229
LOCATION        1     8 144.6542  <.0001

# Relative cover P. mezianum
PMRC=lme(asin(sqrt(PMRC/100))~COMB*LOCATION,data = km0708w,random=~1|BLOCK/PLOT,
+control=lmeControl(returnObject=TRUE),na.action=na.omit)
anova(PMRC)
              numDF denDF   F-value p-value
(Intercept)       1     7 122.62368  <.0001
COMB              1     5   2.36876  0.1844
LOCATION          1     7  82.05754  <.0001
COMB:LOCATION     1     7   0.11052  0.7493

##Dropping the interaction term:
PMRC=lme(asin(sqrt(PMRC/100))~COMB+LOCATION,data = km0708w,random=~1|BLOCK/PLOT,
+control=lmeControl(returnObject=TRUE),na.action=na.omit)
anova(PMRC)
            numDF denDF   F-value p-value
(Intercept)     1     8 122.62321  <.0001
COMB            1     5   2.54274  0.1717
LOCATION        1     8  88.08445  <.0001

# Relative cover L. nutans
LNRC=lme(asin(sqrt(LNRC/100))~COMB*LOCATION,data = km0708w,random=~1|BLOCK/PLOT,
+control=lmeControl(returnObject=TRUE),na.action=na.omit)
anova(LNRC)
              numDF denDF  F-value p-value
(Intercept)       1     7 96.27158  <.0001
COMB              1     5  0.00505  0.9461
LOCATION          1     7 29.70844  0.0010
COMB:LOCATION     1     7  0.00403  0.9512

##Dropping the interaction term:
LNRC=lme(asin(sqrt(LNRC/100))~COMB+LOCATION,data = km0708w,random=~1|BLOCK/PLOT,
+control=lmeControl(returnObject=TRUE),na.action=na.omit)
anova(LNRC)
            numDF denDF   F-value p-value
(Intercept)     1     8 103.11844  <.0001
COMB            1     5   0.00541  0.9442
LOCATION        1     8  31.82132  0.0005

# Relative cover B. insculpta
BIRC=lme(asin(sqrt(BIRC/100))~COMB*LOCATION,data = km0708w,random=~1|BLOCK/PLOT,
+control=lmeControl(returnObject=TRUE),na.action=na.omit)
anova(BIRC)
              numDF denDF   F-value p-value
(Intercept)       1     7  5.858764  0.0461
COMB              1     5  0.003844  0.9530
LOCATION          1     7 22.382267  0.0021
COMB:LOCATION     1     7  0.155355  0.7052
```

```
##Dropping the interaction term:
BIRC=lme(asin(sqrt(BIRC/100))~COMB+LOCATION,data = km0708w,random=~1|BLOCK/PLOT,
+control=lmeControl(returnObject=TRUE),na.action=na.omit)
anova(BIRC)
            numDF denDF   F-value p-value
(Intercept)     1     8  5.858764  0.0418
COMB            1     5  0.004111  0.9514
LOCATION        1     8 23.937556  0.0012

# Relative cover Pseudognaphalium sp.
HELIRC=lme(asin(sqrt(HELIRC/100))~COMB*LOCATION,data = km0708w,random=~1|BLOCK/PLOT,
+control=lmeControl(returnObject=TRUE),na.action=na.omit)
anova(HELIRC)
              numDF denDF  F-value p-value
(Intercept)       1     7 49.36771  0.0002
COMB              1     5  1.88299  0.2284
LOCATION          1     7  0.00057  0.9816
COMB:LOCATION     1     7  3.29484  0.1124

##Dropping the interaction term:
HELIRC=lme(asin(sqrt(HELIRC/100))~COMB+LOCATION,data = km0708w,random=~1|BLOCK/PLOT,
+control=lmeControl(returnObject=TRUE),na.action=na.omit)
anova(HELIRC)
            numDF denDF  F-value p-value
(Intercept)     1     8 44.78880  0.0002
COMB            1     5  1.62331  0.2586
LOCATION        1     8  0.00049  0.9828

# Relative bites on grasses
TGRB=lme(asin(sqrt(TG_RB/100))~COMB*LOCATION,data = km0708w,random=~1|BLOCK/PLOT,
+control=lmeControl(returnObject=TRUE),na.action=na.omit)
anova(TGRB)
              numDF denDF   F-value p-value
(Intercept)       1    15 25924.191  <.0001
COMB              1    14     1.454  0.2478
LOCATION          1    15     7.066  0.0179
COMB:LOCATION     1    15     0.675  0.4241

##Dropping the interaction term:
TGRB=lme(asin(sqrt(TG_RB/100))~COMB+LOCATION,data = km0708w,random=~1|BLOCK/PLOT,
+control=lmeControl(returnObject=TRUE),na.action=na.omit)
anova(TGRB)
            numDF denDF   F-value p-value
(Intercept)     1    16 26047.691  <.0001
COMB            1    14     1.461  0.2467
LOCATION        1    16     7.179  0.0165

# Relative bites B. lachnantha
BLRB=lme(asin(sqrt(BL_RB/100))~COMB*LOCATION,data = km0708w,random=~1|BLOCK/PLOT,
+control=lmeControl(returnObject=TRUE),na.action=na.omit)
anova(BLRB)
              numDF denDF   F-value p-value
(Intercept)       1    15 293.05937  <.0001
COMB              1    14   0.67520  0.4250
LOCATION          1    15 124.58445  <.0001
COMB:LOCATION     1    15   0.33464  0.5715

##Dropping the interaction term:
BLRB=lme(asin(sqrt(BL_RB/100))~COMB+LOCATION,data = km0708w,random=~1|BLOCK/PLOT,
+control=lmeControl(returnObject=TRUE),na.action=na.omit)
anova(BLRB)
            numDF denDF  F-value p-value
(Intercept)     1    16 294.3125  <.0001
COMB            1    14   0.6848  0.4218
LOCATION        1    16 129.3568  <.0001

# Relative bites T. trianrda
TTRB=lme(asin(sqrt(TT_RB/100))~COMB*LOCATION,data = km0708w,random=~1|BLOCK/PLOT,
+control=lmeControl(returnObject=TRUE),na.action=na.omit)
```

```
anova(TTRB)
              numDF denDF   F-value p-value
(Intercept)       1    15  83.53043  <.0001
COMB              1    14   0.06159  0.8076
LOCATION          1    15 111.21933  <.0001
COMB:LOCATION     1    15   4.57686  0.0493

##Post hoc
km0708w$HL=interaction(km0708w$COMB,km0708w$LOCATION)
HL_TTRB=lme(asin(sqrt(TT_RB/100))~-1+HL,data = km0708w,random=~1|BLOCK/PLOT,
+control=lmeControl(returnObject=TRUE),na.action=na.omit)
summary(glht(HL_TTRB, linfct = mcp(HL = "Tukey", interaction_average=TRUE)))

         Simultaneous Tests for General Linear Hypotheses

Multiple Comparisons of Means: Tukey Contrasts


Fit: lme.formula(fixed = asin(sqrt(TT_RB/100)) ~ -1 + HL, data = km0708w,
    random = ~1 | BLOCK/PLOT, na.action = na.omit, control = lmeControl(returnObject = TRUE))

Linear Hypotheses:
                       Estimate Std. Error z value Pr(>|z|)
C+W.OFF - C.OFF == 0    0.06431    0.05528   1.163    0.647
C.TMD - C.OFF == 0     -0.28397    0.06383  -4.449   <1e-04 ***
C+W.TMD - C.OFF == 0   -0.38820    0.05614  -6.915   <1e-04 ***
C.TMD - C+W.OFF == 0   -0.34828    0.05528  -6.301   <1e-04 ***
C+W.TMD - C+W.OFF == 0 -0.45251    0.04618  -9.798   <1e-04 ***
C+W.TMD - C.TMD == 0   -0.10423    0.05614  -1.857    0.244
---
Signif. codes:  0 '***' 0.001 '**' 0.01 '*' 0.05 '.' 0.1 ' ' 1
(Adjusted p values reported -- single-step method)

# Relative bites P. stramineum
PSRB=lme(asin(sqrt(PS_RB/100))~COMB*LOCATION,data = km0708w,random=~1|BLOCK/PLOT,
+control=lmeControl(returnObject=TRUE),na.action=na.omit)
anova(PSRB)
              numDF denDF   F-value p-value
(Intercept)       1    15 237.04248  <.0001
COMB              1    14   0.71762  0.4112
LOCATION          1    15 180.97027  <.0001
COMB:LOCATION     1    15   0.54425  0.4721

##Dropping the interaction term:
PSRB=lme(asin(sqrt(PS_RB/100))~COMB+LOCATION,data = km0708w,random=~1|BLOCK/PLOT,
+control=lmeControl(returnObject=TRUE),na.action=na.omit)
anova(PSRB)
            numDF denDF   F-value p-value
(Intercept)     1    16 236.53756  <.0001
COMB            1    14   0.72911  0.4075
LOCATION        1    16 183.78311  <.0001

# Relative bites P. mezianum
PMRB=lme(asin(sqrt(PM_RB/100))~COMB*LOCATION,data = km0708w,random=~1|BLOCK/PLOT,
+control=lmeControl(returnObject=TRUE),na.action=na.omit)
anova(PMRB)
              numDF denDF  F-value p-value
(Intercept)       1    15 28.57743  0.0001
COMB              1    14  0.00518  0.9436
LOCATION          1    15 35.88106  <.0001
COMB:LOCATION     1    15  0.01418  0.9068

##Dropping the interaction term:
PMRB=lme(asin(sqrt(PM_RB/100))~COMB+LOCATION,data = km0708w,random=~1|BLOCK/PLOT,
+control=lmeControl(returnObject=TRUE),na.action=na.omit)
anova(PMRB)
            numDF denDF  F-value p-value
(Intercept)     1    16 28.65966  0.0001
COMB            1    14  0.00524  0.9433
LOCATION        1    16 37.07918  <.0001
```

```
# Relative bites L. nutans
LNRB=lme(asin(sqrt(LN_RB/100))~COMB*LOCATION,data = km0708w,random=~1|BLOCK/PLOT,
+control=lmeControl(returnObject=TRUE),na.action=na.omit)
anova(LNRB)
              numDF denDF  F-value p-value
(Intercept)       1    15 93.45564  <.0001
COMB              1    14  0.02380  0.8796
LOCATION          1    15 22.82497  0.0002
COMB:LOCATION     1    15  0.74512  0.4016

##Dropping the interaction term:
LNRB=lme(asin(sqrt(LN_RB/100))~COMB+LOCATION,data = km0708w,random=~1|BLOCK/PLOT,
+control=lmeControl(returnObject=TRUE),na.action=na.omit)
anova(LNRB)
            numDF denDF  F-value p-value
(Intercept)     1    16 92.23540  <.0001
COMB            1    14  0.02388  0.8794
LOCATION        1    16 23.03559  0.0002

# Relative bites B. insculpta
BIRB=lme(asin(sqrt(BI_RB/100))~COMB*LOCATION,data = km0708w,random=~1|BLOCK/PLOT,
+control=lmeControl(returnObject=TRUE),na.action=na.omit)
anova(BIRB)
              numDF denDF  F-value p-value
(Intercept)       1    15 10.79044  0.0050
COMB              1    14  6.64232  0.0219
LOCATION          1    15 43.65423  <.0001
COMB:LOCATION     1    15  1.94680  0.1832

##Dropping the interaction term:
BIRB=lme(asin(sqrt(BI_RB/100))~COMB+LOCATION,data = km0708w,random=~1|BLOCK/PLOT,
+control=lmeControl(returnObject=TRUE),na.action=na.omit)
anova(BIRB)
            numDF denDF  F-value p-value
(Intercept)     1    16 11.00681  0.0044
COMB            1    14  6.43419  0.0237
LOCATION        1    16 42.25335  <.0001

# SI grasses
TGSI=lme(TGSI~COMB*LOCATION,data = km0708w,random=~1|BLOCK/PLOT,
+control=lmeControl(returnObject=TRUE),na.action=na.omit, weights = varIdent(form =~ 1 | COMB))
anova(TGSI)
              numDF denDF  F-value p-value
(Intercept)       1     7 578.2308  <.0001
COMB              1     5   2.1553  0.2020
LOCATION          1     7  10.6790  0.0137
COMB:LOCATION     1     7   0.6054  0.4620

##Dropping the interaction term:
TGSI=lme(TGSI~COMB+LOCATION,data = km0708w,random=~1|BLOCK/PLOT,
+control=lmeControl(returnObject=TRUE),na.action=na.omit, weights = varIdent(form =~ 1 | COMB))
anova(TGSI)
            numDF denDF  F-value p-value
(Intercept)     1     8 578.3892  <.0001
COMB            1     5   2.3361  0.1869
LOCATION        1     8  10.6485  0.0115

# SI forbs
TFSI=lme(TFSI~COMB*LOCATION,data = km0708w,random=~1|BLOCK/PLOT,
+control=lmeControl(returnObject=TRUE),na.action=na.omit, weights =varIdent(form =~ 1 | COMB))
anova(TFSI)
              numDF denDF  F-value p-value
(Intercept)       1     7 579.0509  <.0001
COMB              1     5   1.6131  0.2599
LOCATION          1     7   9.7230  0.0169
COMB:LOCATION     1     7   0.5838  0.4698

##Dropping the interaction term:
TFSI=lme(TFSI~COMB+LOCATION,data = km0708w,random=~1|BLOCK/PLOT,
```

```
+control=lmeControl(returnObject=TRUE),na.action=na.omit, weights = varIdent(form =~ 1 | COMB))
anova(TFSI)
            numDF denDF  F-value p-value
(Intercept)     1     8 579.2781  <.0001
COMB            1     5   1.7567  0.2424
LOCATION        1     8   9.6928  0.0144

# SI B. lachnantha
BLSI=lme(BLSI~COMB*LOCATION,data = km0708w,random=~1|BLOCK/PLOT,
+control=lmeControl(returnObject=TRUE),na.action=na.omit)
anova(BLSI)
              numDF denDF  F-value p-value
(Intercept)       1     5 40.09138  0.0014
COMB              1     5  4.85890  0.0787
LOCATION          1     5  0.68192  0.4465
COMB:LOCATION     1     5  0.29168  0.6123

##Dropping the interaction term:
BLSI=lme(BLSI~COMB+LOCATION,data = km0708w,random=~1|BLOCK/PLOT,
+control=lmeControl(returnObject=TRUE),na.action=na.omit)
anova(BLSI)
            numDF denDF  F-value p-value
(Intercept)     1     6 41.00644  0.0007
COMB            1     5  5.17453  0.0720
LOCATION        1     6  0.72064  0.4285

# SI T. triandra
TTSI=lme(TTSI~COMB*LOCATION,data = km0708w,random=~1|BLOCK/PLOT,
+control=lmeControl(returnObject=TRUE),na.action=na.omit)
anova(TTSI)
              numDF denDF   F-value p-value
(Intercept)       1     5 2.6961868  0.1615
COMB              1     5 0.4448440  0.5343
LOCATION          1     4 0.1160899  0.7505
COMB:LOCATION     1     4 1.1622644  0.3417

##Dropping the interaction term:
TTSI=lme(TTSI~COMB+LOCATION,data = km0708w,random=~1|BLOCK/PLOT,
+control=lmeControl(returnObject=TRUE),na.action=na.omit)
anova(TTSI)
            numDF denDF   F-value p-value
(Intercept)     1     5 2.9299803  0.1476
COMB            1     5 0.4433391  0.5350
LOCATION        1     5 0.1023456  0.7620


# SI P. stramineum
PSSI=lme(PSSI~COMB*LOCATION,data = km0708w,random=~1|BLOCK/PLOT,
+control=lmeControl(returnObject=TRUE),na.action=na.omit)
anova(PSSI)
              numDF denDF   F-value p-value
(Intercept)       1     7 1.4664211  0.2652
COMB              1     5 0.0436281  0.8428
LOCATION          1     7 0.1285030  0.7306
COMB:LOCATION     1     7 0.5640142  0.4771

## Dropping the interaction term:
PSSI=lme(PSSI~COMB+LOCATION,data = km0708w,random=~1|BLOCK/PLOT,
+control=lmeControl(returnObject=TRUE),na.action=na.omit)
anova(PSSI)
            numDF denDF   F-value p-value
(Intercept)     1     8 1.5103197  0.2540
COMB            1     5 0.0449341  0.8405
LOCATION        1     8 0.1323499  0.7254

##Dropping the interaction term:
PSSI=lme(PSSI~COMB+LOCATION,data = km0708w,random=~1|BLOCK/PLOT,
+control=lmeControl(returnObject=TRUE),na.action=na.omit)
anova(PSSI)
```

```
# SI P. mezianum
PMSI=lme(PMSI~COMB*LOCATION,data = km0708w,random=~1|BLOCK,
+control=lmeControl(returnObject=TRUE),na.action=na.omit)
anova(PMSI)
Error in MEEM(object, conLin, control$niterEM) :
  Singularity in backsolve at level 0, block 1
##Lack of sufficient data points for mound

##Analysis for off-mound only:
km0708wOFF <- subset(km0708w, YEAR=="2007" & LOCATION=="OFF",select=YEAR:TVGL2BS)
PMSI=lme(PMSI~COMB,data = km0708wOFF,random=~1|BLOCK,control=lmeControl(returnObject=TRUE),
+na.action=na.omit)
anova(PMSI)
            numDF denDF   F-value p-value
(Intercept)     1     5 118.87764  0.0001
COMB            1     5   1.01142  0.3607

# SI L. nutans
LNSI=lme(LNSI~COMB*LOCATION,data = km0708w,random=~1|BLOCK/PLOT,
+control=lmeControl(returnObject=TRUE),na.action=na.omit)
anova(LNSI)
              numDF denDF  F-value p-value
(Intercept)       1     5 5.753426  0.0617
COMB              1     5 1.092700  0.3438
LOCATION          1     3 0.006951  0.9388
COMB:LOCATION     1     3 1.172729  0.3581

##Dropping the interaction term:
LNSI=lme(LNSI~COMB+LOCATION,data = km0708w,random=~1|BLOCK/PLOT,
+control=lmeControl(returnObject=TRUE),na.action=na.omit)
anova(LNSI)
            numDF denDF  F-value p-value
(Intercept)     1     5 7.157964  0.0441
COMB            1     5 0.962181  0.3717
LOCATION        1     4 0.000529  0.9828

# SI B. insculpta
BISI=lme(BISI~COMB*LOCATION,data = km0708w,random=~1|BLOCK/PLOT,
+control=lmeControl(returnObject=TRUE),na.action=na.omit)
anova(BISI)
              numDF denDF  F-value p-value
(Intercept)       1     5 0.707304  0.4387
COMB              1     5 3.568299  0.1175
LOCATION          1     2 0.163756  0.7249
COMB:LOCATION     1     2 0.629877  0.5106

##Dropping the interaction term:
BISI=lme(BISI~COMB+LOCATION,data = km0708w,random=~1|BLOCK/PLOT,
+control=lmeControl(returnObject=TRUE),na.action=na.omit)
anova(BISI)
            numDF denDF  F-value p-value
(Intercept)     1     5 0.715533  0.4362
COMB            1     5 3.505659  0.1201
LOCATION        1     3 0.190586  0.6919

#One sample t-tests

##Total grasses - on-mound
km0708wTMD <- subset(km0708w, LOCATION=="TMD", select=YEAR:TGPCLG)
t.test(km0708wTMD$TGSI, mu=0)

        One Sample t-test

data:  km0708wTMD$TGSI
t = 3.1617, df = 8, p-value = 0.01336
alternative hypothesis: true mean is not equal to 0
95 percent confidence interval:
 0.1812015 1.1578504
sample estimates:
mean of x
```

```
0.6695259

##Total grasses - off-mound
km0708wOFF <- subset(km0708w, LOCATION=="OFF", select=YEAR:TGPCLG)
t.test(km0708wOFF$TGSI, mu=0)

        One Sample t-test

data:  km0708wOFF$TGSI
t = 9.5721, df = 8, p-value = 1.175e-05
alternative hypothesis: true mean is not equal to 0
95 percent confidence interval:
 0.4644388 0.7592305
sample estimates:
mean of x
0.6118347

##Total forbs - on-mound
km0708wTMD <- subset(km0708w, LOCATION=="TMD", select=YEAR:TGPCLG)
t.test(km0708wTMD$TFSI, mu=0)

        One Sample t-test

data:  km0708wTMD$TFSI
t = -3.1964, df = 8, p-value = 0.01268
alternative hypothesis: true mean is not equal to 0
95 percent confidence interval:
 -1.1771608 -0.1904833
sample estimates:
 mean of x
-0.6838221

##Total forbs - off-mound
km0708wOFF <- subset(km0708w, LOCATION=="OFF", select=YEAR:TGPCLG)
t.test(km0708wOFF$TFSI, mu=0)

        One Sample t-test

data:  km0708wOFF$TFSI
t = -10.871, df = 8, p-value = 4.535e-06
alternative hypothesis: true mean is not equal to 0
95 percent confidence interval:
 -0.7732058 -0.5025805
sample estimates:
 mean of x
-0.6378932

##B. lachnantha - on-mound
km0708wTMD <- subset(km0708w, LOCATION=="TMD", select=YEAR:TVPCLG)
t.test(km0708wTMD$BLSI, mu=0)

        One Sample t-test

data:  km0708wTMD$BLSI
t = 5.2323, df = 6, p-value = 0.001953
alternative hypothesis: true mean is not equal to 0
95 percent confidence interval:
 0.3487198 0.9614094
sample estimates:
mean of x
0.6550646

##B. lachnantha - off-mound
km0708wOFF <- subset(km0708w, LOCATION=="OFF", select=YEAR:TVPCLG)
t.test(km0708wOFF$BLSI, mu=0)

        One Sample t-test

data:  km0708wOFF$BLSI
t = 8.8791, df = 8, p-value = 2.047e-05
```

```
alternative hypothesis: true mean is not equal to 0
95 percent confidence interval:
 0.4011624 0.6826360
sample estimates:
mean of x
0.5418992


##T. triandra - on-mound
km0708wTMD <- subset(km0708w, LOCATION=="TMD", select=YEAR:TVPCLG)
t.test(km0708wTMD$TTSI, mu=0)

        One Sample t-test

data:  km0708wTMD$TTSI
t = -0.95285, df = 5, p-value = 0.3844
alternative hypothesis: true mean is not equal to 0
95 percent confidence interval:
 -1.1433996  0.5249753
sample estimates:
 mean of x
-0.3092121

##T. triandra - off-mound
km0708wOFF <- subset(km0708w, LOCATION=="OFF", select=YEAR:TVPCLG)
t.test(km0708wOFF$TTSI, mu=0)

        One Sample t-test

data:  km0708wOFF$TTSI
t = -3.3588, df = 8, p-value = 0.009949
alternative hypothesis: true mean is not equal to 0
95 percent confidence interval:
 -0.32717307 -0.06080532
sample estimates:
 mean of x
-0.1939892

## P. stramineum - on-mound
km0708wTMD <- subset(km0708w, LOCATION=="TMD", select=YEAR:TVPCLG)
t.test(km0708wTMD$PSSI, mu=0)

        One Sample t-test

data:  km0708wTMD$PSSI
t = -0.48127, df = 8, p-value = 0.6432
alternative hypothesis: true mean is not equal to 0
95 percent confidence interval:
 -0.5884925  0.3852670
sample estimates:
 mean of x
-0.1016127

## P. stramineum - off-mound
km0708wOFF <- subset(km0708w, LOCATION=="OFF", select=YEAR:TVPCLG)
t.test(km0708wOFF$PSSI, mu=0)

        One Sample t-test

data:  km0708wOFF$PSSI
t = -2.1885, df = 8, p-value = 0.06006
alternative hypothesis: true mean is not equal to 0
95 percent confidence interval:
 -0.38418615  0.01004669
sample estimates:
 mean of x
-0.1870697

## P. mezianum - on-mound
km0708wTMD <- subset(km0708w, LOCATION=="TMD", select=YEAR:TVPCLG)
```

```
t.test(km0708wTMD$PMSI, mu=0)

        One Sample t-test

data:  km0708wTMD$PMSI
t = -1.5063, df = 2, p-value = 0.271
alternative hypothesis: true mean is not equal to 0
95 percent confidence interval:
 -2.317759  1.115759
sample estimates:
 mean of x
-0.6009999

## P. mezianum - off-mound
km0708wOFF <- subset(km0708w, LOCATION=="OFF", select=YEAR:TGPCLG)
t.test(km0708wOFF$PMSI, mu=0)

        One Sample t-test

data:  km0708wOFF$PMSI
t = -17.147, df = 8, p-value = 1.36e-07
alternative hypothesis: true mean is not equal to 0
95 percent confidence interval:
 -0.8042682 -0.6135933
sample estimates:
 mean of x
-0.7089308

## L. nutans - on-mound
km0708wTMD <- subset(km0708w, LOCATION=="TMD", select=YEAR:TVPCLG)
t.test(km0708wTMD$LNSI, mu=0)

        One Sample t-test

data:  km0708wTMD$LNSI
t = -0.85916, df = 4, p-value = 0.4387
alternative hypothesis: true mean is not equal to 0
95 percent confidence interval:
 -1.5032100  0.7927393
sample estimates:
 mean of x
-0.3552354

## L. nutans - off-mound
km0708wOFF <- subset(km0708w, LOCATION=="OFF", select=YEAR:TVPCLG)
t.test(km0708wOFF$LNSI, mu=0)

        One Sample t-test

data:  km0708wOFF$LNSI
t = -13.041, df = 8, p-value = 1.135e-06
alternative hypothesis: true mean is not equal to 0
95 percent confidence interval:
 -0.4753520 -0.3325018
sample estimates:
 mean of x
-0.4039269

## B. insculpta - on-mound
km0708wTMD <- subset(km0708w, LOCATION=="TMD", select=YEAR:TVPCLG)
t.test(km0708wTMD$BISI, mu=0)

        One Sample t-test

data:  km0708wTMD$BISI
t = 0, df = 3, p-value = 1
alternative hypothesis: true mean is not equal to 0
95 percent confidence interval:
 -1.837386  1.837386
sample estimates:
```

```
mean of x
        0

## B. insculpta - off-mound
km0708wOFF <- subset(km0708w, LOCATION=="OFF", select=YEAR:TVPCLG)
t.test(km0708wOFF$BISI, mu=0)

        One Sample t-test

data:  km0708wOFF$BISI
t = -1.2842, df = 8, p-value = 0.235
alternative hypothesis: true mean is not equal to 0
95 percent confidence interval:
 -0.6622944  0.1885053
sample estimates:
 mean of x
-0.2368945


#PERMANOVA and NMDS
library(vegan)
library(RColorBrewer)

#read in all vegetation and bite data
cattle<-read.csv("veg_data_off_on_mound.csv")
CV<-read.csv("bites_veg_combined.csv")

##Differences between plant communities on and off termite mounds
and in different herbivore treatments####

  #prepare data
  CV1<-CV[CV$Type=="Veg",]
  env<-CV1[,c(1:8)]
  veg<-CV1[,c(8:45)]


  rownames(veg) <- veg$Plot.1
  rownames(env) <- env$Plot.1
  veg$Plot.1 <- NULL
  env$Plot.1 <- NULL
  veg<-sapply(veg, as.numeric)

  #Create distance matrix for plants vs. plots.
Run PERMANOVA based on bray-curtis distance. Stratify data by Block.
Location refers to on vs. off mound. Wildlife refers to herbivore treatment####

  vegdist1 <- vegdist(veg,method = "bray" )
  adonis(vegdist1 ~ LOCATION + Wildlife , strata = env$Block,
+data = env, contr.unordered = "contr.sum", permutations = 1000)

##Differences between plants cattle consumed on and off termite mounds
and in different herbivore treatments####

  CV2<-CV[CV$Type=="Bite",]
  env<-CV2[,c(1:8)]
  veg<-CV2[,c(8:45)]


  rownames(veg) <- veg$Plot.1
  rownames(env) <- env$Plot.1
  veg$Plot.1 <- NULL
  env$Plot.1 <- NULL
  veg<-sapply(veg, as.numeric)


  vegdist1 <- vegdist(veg,method = "bray" )
  adonis(vegdist1 ~ LOCATION + Wildlife , strata = env$BLOCK,
+data = env, contr.unordered = "contr.sum", permutations = 1000)
```

```
##NMDS visualization of differences between plant communities and plants
cattle consumed on and off termite mounds####

  env<-CV[,c(1:8)]
  veg<-CV[,c(8:45)]
  rownames(veg) <- veg$Plot.1
  rownames(env) <- env$Plot.1
  veg$Plot.1 <- NULL
  env$Plot.1 <- NULL
  veg<-sapply(veg, as.numeric)

  #create distance matrix for all vegetation and bite data
  vegNMDS <- metaMDS(veg, distance = "bray")

  #graph NMDS of termite mound location (on vs. off) X data collection
(bites vs. vegetation)
  colors<-brewer.pal(4,"Pastel2")
  plot(vegNMDS, display = c("site"), type = "n" , xlim = c(-1.5,2),ylim = c(-2,1.5))
  with(env, points(vegNMDS, display = "sites", col = colors[Loc_type],
+pch = 21, bg = colors[Loc_type]))
  with(env, legend(x = 1.9, y = 1.5, legend = levels(Loc_type),col = colors,
+pch = 21, pt.bg = colors))

  for(i in 1:4) {

    ordiellipse(vegNMDS, group=env$Loc_type, kind="sd", conf=0.95, label=T, font=1, cex=.70,
+draw = "polygon", col=colors[i],show.groups=groupz[i])

  }
```
